# Joint association of aerobic physical activity and muscle-strengthening activities with metabolic syndrome : the Korean National Health and Nutrition Examination Survey 2014-2015

**DOI:** 10.4178/epih.e2021096

**Published:** 2021-11-06

**Authors:** Jungjun Lim, Soyoung Park, Joon-Sik Kim

**Affiliations:** Department of Physical Education, College of Education, Seoul National University, Seoul, Korea

**Keywords:** Physical activity, Strength training, Metabolic syndrome, Epidemiology

## Abstract

**OBJECTIVES:**

The study aimed to examine whether simultaneously meeting the combined guidelines of accelerometer-assessed moderate to vigorous physical activity (MVPA) and self-reported muscle-strengthening activity (MSA) was associated with lower odds of metabolic syndrome (MetS) than meeting neither or 1 of the guidelines among the Koreans.

**METHODS:**

This cross-sectional analysis included 1,355 participants from the Korea National Health and Nutrition Examination Survey (2014-2015). Logistic regression was used to analyze the associations across groups of MVPA-MSA guideline adherence (meeting neither [reference]; meeting MVPA only; meeting MSA only; meeting both MVPS and MSA) with MetS components (abdominal obesity, hypertriglyceridemia, low high-density lipoprotein cholesterol [HDL-C], hypertension, and hyperglycemia). The odds ratios (ORs) were adjusted for covariates (e.g., sex, age, body mass index, and accelerometer wearing time).

**RESULTS:**

MSA only significantly reduced the OR for abdominal obesity (OR, 0.34; 95% confidence interval [CI], 0.13 to 0.91). Meeting both MVPA and MSA reduced the OR for hypertriglyceridemia (OR, 0.59; 95% CI, 0.39 to 0.88) and low HDL-C (OR, 0.46; 95% CI, 0.31 to 0.68). Compared to meeting neither, MVPA only (OR, 0.63; 95% CI, 0.44 to 0.89) and both MVPA and MSA (OR, 0.46; 95% CI, 0.28 to 0.76) significantly reduced the OR for MetS.

**CONCLUSIONS:**

Combined MVPA-MSA was more beneficially associated with MetS prevalence than MVPA only and MSA only. Considering that more than 85% of Korean adults do not meet both the MVPA and MSA guidelines, public health actions to promote adherence should be supported.

## INTRODUCTION

Metabolic syndrome (MetS) is a collection of risk factors associated with cardiovascular disease and type 2 diabetes mellitus. MetS is defined as including high blood pressure (BP), high cholesterol, elevated abdominal adiposity, and abnormal fasting plasma glucose [1,2]. Increased levels of MetS risk factors are closely associated with mortality [3] and morbidity [4], as well as complications such as stroke, myocardial infarction, and angina [5]. The worldwide prevalence of MetS is estimated to be 20-25% among adults [6,7]. Similarly, the prevalence of MetS in Korea has steadily increased from 22.6% in 2013 to 30.4% in 2018 [8], and the high prevalence of MetS is considered a significant public health concern.

The association between physical activity (PA) and metabolic health-related outcomes has been studied extensively [9-11]. For the prevention and treatment of MetS, the World Health Organization (WHO) recommends that adults should participate in aerobic moderate-to-vigorous physical activity (MVPA) and follow the muscle-strengthening activity (MSA) guidelines (≥ 150 min/wk of MVPA and ≥ 2 session/wk of MSA) [12]. Given that these guidelines distinguish between aerobic MVPA and MSA, these types of exercise may promote beneficial adaptations in particular physiological systems that improve the risk associated with a variety of MetS-related components. Despite the reported benefits of various types of PA for MetS, epidemiological studies have mostly discussed the benefits of MVPA [13,14]. However, over the last decade, emerging evidence has suggested that MSA can independently ameliorate or prevent MetS-related risk factors [15,16].

Although there are global recommendations on both types of activities (MVPA and MSA) and the benefits of each type, there is a paucity of studies examining the association between combined MVPA-MSA and MetS [4]. Some studies have indicated that concurrently meeting the MVPA-MSA guidelines had the strongest association with MetS prevalence, as compared to meeting the individual guidelines for MVPA and MSA alone or meeting neither of them [17].

Among United States adults with hypertension (n= 155,791), high cholesterol (n= 141,173), and diabetes (n= 50,027), the lowest prevalence ratios were seen in those who met the combined MVPA-MSA guidelines, followed by those who met the guidelines for MSA only, MVPA only, and met neither of the 2 guidelines (reference group) [18]. Despite the finding that meeting both MVPA and MSA recommendations had the strongest positive effect on MetS-related risk factors, most studies have been conducted mainly among Caucasians [4]. Therefore, whether these beneficial results can be generalized globally remains unclear. Given the possibility that associations between cardiometabolic risk factors may be dissimilar among ethnicities [19,20], it is necessary to examine these associations in other ethnic groups, such as Asians. Moreover, although objective accelerometer-assessed MVPA showed a stronger association with MetS prevalence than self-reported MVPA [11,21,22], no study has confirmed the association between MetS and simultaneously meeting both the MVPA guidelines, as measured by accelerometry, and the MSA recommendations, as measured by self-reporting, in Koreans.

Therefore, this study aimed to investigate whether simultaneously meeting the combined guidelines of accelerometer-assessed MVPA and self-reported MSA was associated with lower odds of MetS than meeting neither or 1 of the guidelines among Koreans.

## MATERIALS AND METHODS

### Participants

The data for the study were collected from the 2014-2015 Korea National Health and Nutrition Examination Survey (KNHANES), a nationally representative cross-sectional survey of samples from Korea that was conducted by the Korea Disease Control and Prevention Agency. The KNHANES is designed to collect health and nutritional status information and to monitor trends in health risk factors and the prevalence of major chronic diseases in Korea.

**Figure 1.**
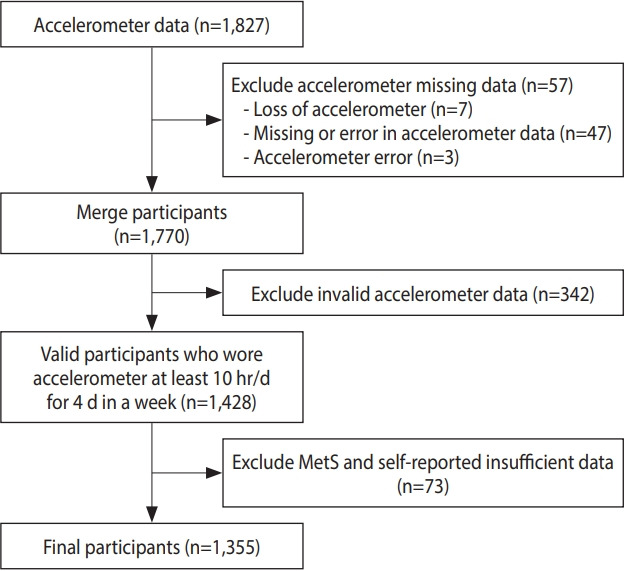
Flow diagram of study participant selection. MetS, metabolic syndrome.

Among the 2014-2015 KNHANES participants, adults who agreed to wear an accelerometer were selected for this analysis (n= 1,827). Participants were excluded if they lost their accelerometer (n= 7), had missing or erroneous accelerometer data (n= 47), had accelerometer errors (n= 3), wore the accelerometer for an insufficient time (n= 342), or had insufficient data on MetS-related risk factors and the MSA questionnaire (n= 73). Finally, a total of 1,355 participants were eligible for this study (Figure 1).

### Accelerometer-assessed physical activity

The accelerometer used in the KNHANES was the wGT3X+ (ActiGraph, Pensacola, FL, USA). The reading on the accelerometer was noted every day for a week from midnight on the day after the examination, and participants were instructed to wear the accelerometer at all possible times, except during water-based activities, such as swimming, bathing, and sleeping [23]. To assess daily PA patterns, individuals wore the accelerometer for at least 10 hours per day for a minimum of 4 days, and they were included in the analysis regardless of whether the data were from weekdays or weekends [24]. To distinguish the accelerometer nonwearing time, a minimum period of 60 consecutive minutes of 0 activity count was defined, with an allowance of 1-2 minutes of activity counts between 0 and 100 [25]. MVPA was defined as > 2,020 activity counts per minute [25]. According to the PA guidelines for Americans, meeting the PA guidelines was defined as accelerometer-determined MVPA of 600 metabolic equivalents of task (MET)-min/wk or more, and every minute of MVPA was used [12].

### Self-reported muscle-strengthening activities

The KNHANES, based on the Global Physical Activity Questionnaire, which is a self-reported questionnaire to survey PA along with the number of days of MSA, was investigated. The question on MSA in the questionnaire was as follows: “In the past week, on how many days did you do push-ups, sit-ups, pull-ups, dumbbells or barbells training designed to strengthen your muscles?” The responses ranged from “not at all” to “more than 5 days” with a weekly frequency. According to the PA guidelines for Americans, meeting the MSA guidelines was defined as engaging in MSA at least 2 days a week [12].

### Metabolic syndrome

The presence of MetS was determined according to the criteria established by the National Cholesterol Education Program Adult Treatment Panel III. However, waist circumference was determined according to the Asian standard criteria [27]. Participants meeting 3 or more of the following five criteria were classified as having MetS: (1) waist circumference ≥ 90 cm in male and ≥ 85 cm in female; (2) serum triglyceride level of ≥ 150 mg/dL; (3) high-density lipoprotein cholesterol (HDL-C) level < 40 mg/dL in male and < 50 mg/dL in female; (4) fasting glucose level ≥ 100 mg/dL; and (5) systolic BP ≥ 130 mmHg and/or diastolic BP ≥ 85 mmHg. Users of medication for dyslipidemia, diabetes, and hypertension were excluded from the study.

### Covariates

In the present study, sex, age, family income, education, alcohol consumption, smoking, body mass index (BMI), and accelerometer wearing time were selected as covariates [28-30]. Age was used as a continuous variable and BMI was classified into 3 groups (underweight: < 18.5 kg/m^2^; normal: 18.5-24.9 kg/m^2^; and obese: ≥ 25.0 kg/m^2^) according to the Asian-Pacific recommendations. Family income was divided into quartiles. Educational level was divided into an elementary school degree or less, middle school degree or less, high school degree or less, and college or higher. Alcohol consumption was classified into 3 categories based on respondents’ reported alcohol consumption: those who never drank, those who consumed 1 drink or less per month in the past 1 year, and those who consumed 1 drink or more per month in the past 1 year. Smoking was divided into past smoking, non-smoking, and current smoking.

### Statistical analysis

Baseline characteristics and the adherence rate to the PA guidelines according to MetS are presented as mean (standard error) for continuous variables and as count and percentage for categorical variables. The chi-square frequency test was used to assess the adherence rate to the PA guidelines concerning MetS. Odds ratios (ORs) and 95% confidence intervals (CIs) were obtained using logistic regression to analyze the associations of meeting only the MVPA guideline, meeting only the MSA guideline, or meeting both the MVPA and MSA guidelines with MetS. Accelerometer raw data processing was performed using SAS version 9.4 (SAS Institute Inc., Cary, NC, USA), and Stata/SE version 12.0 (StataCorp., College Station, TX, USA) was used for statistical analysis. The statistical significance level was established as a p-value of < 0.05.

### Ethics statement

This study protocol was approved by the Institutional Review Board of Seoul National University (IRB No. E2011/001-011). Informed consent was obtained from each participant during the survey.

## RESULTS

The baseline characteristics of the participants are presented in Table 1. The average age of 1,355 adults was 44.7 years, and 63.6% were females. Among the participants, 64.3% had a normal BMI, 31.5% were obese, and 4.1% were underweight. Regarding the PA categories, 37.6% of the participants did not meet the combined or isolated MVPA and MSA guidelines, 41.5% met only the MVPA guideline, 6.0% met only the MSA guideline, and 14.7% met both guidelines. Additionally, in terms of MetS components, 21.1% had abdominal obesity, 30.1% had hypertriglyceridemia, 36.7% had low HDL-C, 27.9% had hypertension, and 27.3% had hyperglycemia.

The adherence rate to the PA guidelines according to MetS are described in Table 2. The proportion of people who did not meet either PA guideline was higher in the group with MetS than in the group without MetS (41.6 vs. 36.4%). However, the proportion of people meeting both PA guidelines was lower in the group with MetS than in the group without MetS (11.7 vs. 15.6%). The MSA-only (with MetS 6.1%; without MetS 6.0%) and MVPA-only (with MetS 40.3%; without MetS 41.8%) groups showed similar proportions of MetS. There was no statistically significant differences in the adherence rate to the PA guidelines concerning MetS.

The associations of PA categories (reference group= met neither guideline) with MetS components (abdominal obesity, hypertriglyceridemia, low HDL-C, hypertension, and hyperglycemia) are described in Table 3. Meeting the MSA guideline only significantly reduced the OR for abdominal obesity (OR, 0.34; 95% CI, 0.13 to 0.91). In contrast, MVPA did not show any statistically significant associations. Nevertheless, meeting the combined MVPA-MSA guidelines reduced the ORs for hypertriglyceridemia and low HDL-C (OR, 0.59; 95% CI, 0.39 to 0.88 and OR, 0.46, 95% CI, 0.31 to 0.68, respectively).

**Figure 2.**
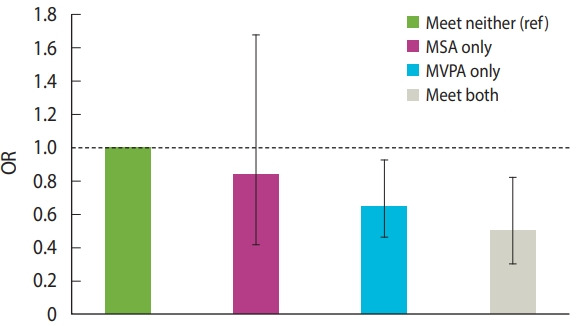
Odds ratios (ORs) with 95% confidence intervals (95% CIs) for metabolic syndrome among individuals meeting MSA and MVPA, and both guidelines. The OR (95% CI) are as follows: MSA only: 0.81 (0.40, 1.62); MVPA only: 0.63 (0.44, 0.89); both: 0.46 (0.28, 0.76). The values were adjusted for the following covariates: sex, age, family income, education, alcohol consumption, smoking, body mass index, and accelerometer wearing time. MSA, muscle-strengthening activity; MVPA, moderate to vigorous physical activity. ref, reference.

The associations of PA categories (reference= met neither guideline) with MetS are shown in Figure 2. Compared to meeting neither guideline, meeting MVPA and both guidelines significantly reduced the OR for MetS (OR, 0.63; 95% CI, 0.44 to 0.89 and OR, 0.46; 95% CI, 0.28 to 0.76, respectively). In contrast, MSA did not show a significant association (OR, 0.81; 95% CI, 0.40 to 1.62).

## DISCUSSION

We investigated for the first time the associations between different combinations of accelerometer-measured MVPA and selfreported MSA guideline compliance with MetS and its components in a large sample of Asian adults. The main finding was that achieving the combined MVPA-MSA guidelines was independently associated with more beneficial MetS-related parameters than meeting neither guideline, the MVPA guideline alone, or the MSA guideline alone. Our key findings show that meeting both the MVPA and MSA guidelines was independently associated with an optimally low MetS prevalence in Korean adults.

Currently, the epidemiological evidence on the association between PA and metabolic health is based on aerobic MVPA guidelines. Both self-reported MVPA and objectively measured MVPA have been reported to exert many positive effects on MetS-related outcomes [31-34]. However, some studies have reported significant low-to-moderate correlations between objectively measured MVPA and self-reported MVPA [35,36]. In particular, objectively measured MVPA using an accelerometer showed much stronger associations with MetS-related components than self-reported MVPA, even after adjusting for several potential confounders [11,22]. Our results also showed that meeting the MVPA guideline only significantly reduced the prevalence of MetS (OR, 0.63; 95% CI, 0.44 to 0.89). This is similar to the results of previous studies on populations in Western countries (OR, 0.39; 95% CI, 0.31 to 0.48) [17]. However, the relationship between meeting the MVPA guideline only and MetS-related components was not statistically significant. These results may be affected by whether the PA was measured objectively or subjectively, statistical techniques, and differences in the study participants (e.g., their medical history and ethnicity). In contrast, compared with studies on the epidemiology of aerobic MVPA, which have been conducted for decades, studies on MSA are scarce. Studies on clinical exercise have shown that MSA is associated with metabolic health indicators (e.g., glucose/lipid metabolism, obesity, and BP) [37,38], skeletal muscle (e.g., bone density and hypertrophy) [39,40], and functional capacity (e.g., balance and performance) [41,42]. However, we showed that meeting the MSA recommendation alone (≥ 2 d/wk) was not associated with MetS-related components other than waist circumference, which supports the findings of another study involving Korean adults [4]. These findings might be explained by the optimal dose, intensity, and frequency of MSA on metabolic health, which remain unclear, and bias due to subjective reporting (e.g., error in recall and overestimation of exercise) that cannot be excluded. In addition, since MSA requires relatively more exercise equipment (dumbbell and exercise machine), exercise skill proficiency (e.g., to perform squats, push-ups, and deadlifts), and understanding of the terminology and principles related to resistance exercises (e.g., set, repeated maximum, progression, and overload), it is judged that achieving the recommendations will be more difficult for MSA than for aerobic MVPA.

Our results provide early insights into the potentially beneficial role of MVPA, MSA, and combined MVPA-MSA on detrimental MetS-related components in a large population-based sample. Our key findings support the extensive literature on the benefits of achieving both aerobic and resistance PA recommendations for metabolic health. In particular, since there was no decrease in abdominal obesity, the reduction in MetS prevalence associated with combined MVPA-MSA appears to have been achieved through the improvement of dyslipidemia. Furthermore, compared with previous studies in other countries, which predominantly included Caucasians as participants, we identified comparable beneficial MetS-related components for meeting combined MVPA-MSA recommendations [17]. Our findings support the findings of Bennie et al. [4], who measured MVPA subjectively in Korean adults, and a longitudinal study showing that achieving both MVPA and MSA recommendations was associated with a lower risk of mortality than achieving 1 recommendation [43]. These positive physiological effects may be explained by the fact that the combined effects of both MVPA and MSA can be greater than their individual effects [44].

In our study, only 14.7% of Korean adults met the combined MVPA-MSA recommendations. This achievement rate was similar to that reported in Australia (15%) [45], but lower than that in Germany (22.6%) [46] and the United States (20.2%) [47]. Despite the growing recognition of the potential health benefits of combined MVPA-MSA, approximately 80-85% of currently reported sample populations from multiple countries have not met the recommended aerobic MVPA and MSA guidelines. Moreover, as a result of examining the adherence rate to PA guidelines according to the presence or absence of MetS in our study, the MVPA-only and MSA-only categories showed similar PA achievement rates with or without MetS. However, despite the many benefits of combined MVPA-MSA, people with MetS had a lower achievement rate for the combined MVPA-MSA recommendations than those without MetS. Therefore, we need to closely examine the requirements for achieving both MVPA and MSA recommendations and support various strategies to promote adherence to both MVPA and MSA modalities.

Our study has certain limitations. First, because the nature of cross-sectional studies makes it difficult to infer causality, the results of this study should be interpreted with caution. Future longitudinal studies on participants who meet both the MVPA and MSA guidelines are needed to clarify the associations between achieving both MVPA and MSA recommendations and MetS-related components. Second, our results were derived from subjects who had PA measurements made using an accelerometer, which may have affected the results. For example, those excluded from the analysis because they did not wear an accelerometer may have been among the most physically inactive participants, which may have affected the results in relatively unpredictable ways. Lastly, MSA was measured using self-reported questionnaires. Although the validity of this questionnaire has been proven, it may have been influenced by bias (e.g., over-estimated PA dose and recall bias). In addition, the MSA questionnaire lacks some information about the frequency, intensity, and time of participation compared to the MVPA questionnaire; this information might be required for a more detailed analysis.

## Figures and Tables

**Table 1.** Characteristics of participants

Characteristics	Mean (SE) or n (%)	95% CI	
		Lower limit	Upper limit
Age (yr)	44.7 (0.3)	44.1	45.4
Sex (female)	862 (63.6)	61.0	66.1
Educational level			
<Elementary school	112 (8.2)	6.7	9.7
<Middle school	135 (9.9)	8.3	11.5
<High school	545 (40.2)	37.6	42.8
>Undergraduate	563 (41.5)	38.9	44.1
Family income (percentile)			
<25	91 (6.7)	5.3	8.0
25-50	343 (25.3)	22.9	27.6
50-75	457 (33.7)	31.2	36.2
75-100	464 (34.2)	31.7	36.7
Smoking^1^			
No	1,175 (86.7)	84.9	88.5
Yes	180 (13.2)	11.4	15.0
Alcohol^2^			
No	612 (45.1)	42.5	47.8
Yes	743 (54.8)	52.1	57.4
BMI (kg/m^2^)			
Underweight (<18.5)	56 (4.1)	3.0	5.1
Normal (18.5-24.9)	872 (64.3)	61.8	66.9
Obese (≥25.0)	427 (31.5)	29.0	33.9
Physical activity guideline adherence			
Meet neither	510 (37.6)	35.0	40.2
MVPA only^3^	563 (41.5)	38.9	44.1
MSA only^4^	82 (6.0)	4.7	7.3
Meet both	200 (14.7)	12.8	16.6
Metabolic syndrome	307 (22.6)	20.4	24.8
High waist circumference	287 (21.1)	19.0	23.3
High triglyceride	408 (30.1)	27.6	32.5
Low HDL-cholesterol	498 (36.7)	34.1	39.3
High blood pressure	379 (27.9)	25.5	30.3
High fasting glucose	371 (27.3)	25.0	29.7

SE, standard error; CI, confidence interval; BMI, body mass index, MVPA, moderate to vigorous physical activity, MSA, muscle-strengthening activity, HDL, high-density lipoprotein.

1 Smoking: No means ‘have never smoked cigarettes before’ or ‘past smoker,’ Yes means ‘current smoker.’

2 Alcohol: No means ‘have never consumed alcohol before’ or ‘less than1 glass of alcohol was consumed in a month in a recent year,’ Yes means ‘more than 1 glass of alcohol was consumed in a month in a recent year.’

3 MVPA only: A group that met the MVPA guideline but not the MSA guideline.

4 MSA only: A group that met the MSA guideline but not the MVPA guideline.

**Table 2.** Adherence rate to physical activity guidelines according to MetS

Variables	People with MetS	People without MetS	p-value^1^
Meet neither	128 (41.6)	382 (36.4)	0.095
MSA only	19 (6.1)	63 (6.0)	0.909
MVPA only	124 (40.3)	439 (41.8)	0.639
Meet both	36 (11.7)	164 (15.6)	0.088

Data are presented as number (%). MetS, metabolic syndrome, MSA, muscle-strengthening activity, MVPA, moderate to vigorous physical activity.

1 Chi-square test.

**Table 3.** Adjusted ORs detailing the independent and combined effects of meeting the MSA and MVPA guidelines^1^

MetS risk factors	OR (95% CI)	p-value
High waist circumference		
Met neither	1.00 (reference)	
MSA only	0.34 (0.13, 0.91)	0.032
MVPA only	0.92 (0.58, 1.45)	0.724
Met both	0.87 (0.46, 1.64)	0.673
High triglycerides		
Met neither	1.00 (reference)	
MSA only	0.86 (0.49, 1.51)	0.613
MVPA only	0.82 (0.61, 1.09)	0.179
Met both	0.59 (0.93, 0.88)	0.011
Low HDL-cholesterol		
Met neither	1.00 (reference)	
MSA only	0.90 (0.54, 1.50)	0.691
MVPA only	0.84 (0.64, 1.09)	0.191
Met both	0.46 (0.31, 0.68)	<0.001
High blood pressure		
Met neither	1.00 (reference)	
MSA only	1.41 (0.79, 2.25)	0.243
MVPA only	1.27 (0.94, 1.71)	0.117
Met both	1.03 (0.68, 1.57)	0.859
High fasting glucose		
Met neither	1.00 (reference)	
MSA only	1.07 (0.59, 1.93)	0.819
MVPA only	1.03 (0.76, 1.39)	0.816
Met both	0.92 (0.61, 1.40)	0.712

MetS, metabolic syndrome; CI, confidence interval; MSA: muscle-strengthening activity; MVPA: moderate to vigorous physical activity; HDL, high-density lipoprotein.

1 Adjusted for the following covariates: sex, age, family income, education, alcohol consumption, smoking, body mass index, and accelerometer wearing time.
